# Study on diversity, nitrogen-fixing capacity, and heavy metal tolerance of culturable *Pongamia pinnata* rhizobia in the vanadium-titanium magnetite tailings

**DOI:** 10.3389/fmicb.2023.1078333

**Published:** 2023-06-19

**Authors:** Tian Shen, Ruimin Jin, Jing Yan, Xiran Cheng, Lan Zeng, Qiang Chen, Yunfu Gu, Likou Zou, Ke Zhao, Quanju Xiang, Petri Penttinen, Menggen Ma, Shuangcheng Li, Ting Zou, Xiumei Yu

**Affiliations:** ^1^College of Resources, Sichuan Agricultural University, Chengdu, China; ^2^Key Laboratory of Investigation and Monitoring, Protection and Utilization for Cultivated Land Resources, Ministry of Natural Resources, Chengdu, China

**Keywords:** rhizobia, *Pongamia pinnata*, VTM tailings, nitrogen fixation, plant growth promotion, tolerance

## Abstract

**Introduction:**

The diversity, nitrogen-fixing capacity and heavy metal tolerance of culturable rhizobia in symbiotic relationship with *Pongamia pinnata* surviving in vanadium (V) - titanium (Ti) magnetite (VTM) tailings is still unknown, and the rhizobia isolates from the extreme barren VTM tailings contaminated with a variety of metals would provide available rhizobia resources for bioremediation.

**Methods:**

*P. pinnata* plants were cultivated in pots containing the VTM tailings until root nodules formed, and then culturable rhizobia were isolated from root nodules. The diversity, nitrogen-fixing capacity and heavy metal tolerance of rhizobia were performed.

**Results:**

Among 57 rhizobia isolated from these nodules, only twenty strains showed different levels of tolerance to copper (Cu), nickel (Ni), manganese (Mn) and zinc (Zn), especially strains PP1 and PP76 showing high tolerance against these four heavy metals. Based on the phylogenetic analysis of 16S rRNA and four house-keeping genes (*atpD*, *recA*, *rpoB*, *glnII*), twelve isolates were identified as *Bradyrhizobium pachyrhizi*, four as *Ochrobactrum anthropic*, three as *Rhizobium selenitireducens* and one as *Rhizobium pisi*. Some rhizobia isolates showed a high nitrogen-fixing capacity and promoted *P. pinnata* growth by increasing nitrogen content by 10%-145% in aboveground plant part and 13%-79% in the root. *R. pachyrhizi* PP1 showed the strongest capacity of nitrogen fixation, plant growth promotion and resistance to heavy metals, which provided effective rhizobia strains for bioremediation of VTM tailings or other contaminated soils. This study demonstrated that there are at least three genera of culturable rhizobia in symbiosis with *P. pinnata* in VTM tailings.

**Discussion:**

Abundant culturable rhizobia with the capacity of nitrogen fixation, plant growth promotion and resistance to heavy metals survived in VTM tailings, indicating more valuable functional microbes could be isolated from extreme soil environments such as VTM tailings.

## Introduction

Tailings are the part of the product of separation operation in mineral processing that has low content of useful target components and cannot be used for production. Vanadium (V) – titanium (Ti) magnetite (VTM) is a widely distributed mineral ore containing oxides of V, Ti, and Fe, which become large amounts of heavy metal-containing slag after being subjected to the iron and steel smelting process. Because of its physicochemical characteristics, soil often becomes the most direct acceptor of pollutants from the processing of minerals such as titanium and magnetite (Wang et al., 2018; Demková et al., 2019). The soil around a mining or smelting area will continuously accumulate the byproducts of the mining processes, resulting in serious heavy metal pollution, e.g., Cd was determined to be the main heavy metal pollutant in the Dahuangshan mining area (Zeng et al., 2022). Heavy metals are not easily degraded and can persist for years in the soil (He et al., 2019). Plants can absorb metal ions through their roots and invertebrates can ingest metal-containing particles so that they enter the food chain where they may be ingested by larger animals or even humans, harming the environment and endangering human health (Jin et al., 2014). Meanwhile, plant extract remediating metal in contaminated environmental has been considered as sustainable and environmentally friendly way (Upadhyay et al., 2023). Therefore, in order to achieve sustainability in the mining industry, one of the most urgent tasks is to concentrate on the reclamation of land contaminated with mine tailings and soil remediation in mining areas. There are some sustainable measures to deal with unavoidable heavy metal and fly-ash pollution, e. g. arsenic contamination in rice agro-ecosystems is migitated by using biochar, organic fertilizers, nanomaterials (Upadhyay and Edrisi, 2021; Upadhyay et al., 2023). Some emerging methods such as CRISPR and nanotechnological approaches along with PGPR also can manage degraded soil effectively (Upadhyay et al., 2022).

*Pongamia pinnata* is a deep-rooted Asian tree in the *Fabaceae* family, which has strong tolerance to salt, drought and heat, and can withstand submersion in fresh water (Marriboina and Attipalli, 2020). The root system of *P. pinnata* is extensive, and the root nodules are large and numerous with strong nitrogen fixation ability. Kumar et al. (2017) found that *P. pinnata* increased antioxidant and nutrient accumulation to protect plants under heavy metal stress. *P. pinnata* can grow well in the soil polluted with heavy metals and already shows good remediation potential (Yu et al., 2019). These characteristics make *P. pinnata* an excellent pioneer plant for removing heavy metal contaminants from soils (Marriboina and Attipalli, 2020).

As an important nutrition of plant growth, nitrogen is supplied through the biological nitrogen fixation by some endophytic diazotrophs of crops or soil microorganism (Singh et al., 2022). Legume-rhizobium symbiotic system shows strong nitrogen fixation capacity and strong resistance to heavy metal through the mutually beneficial relationship between rhizobia and the host plant (Zhang P. et al., 2019). Rhizobia can increase the heavy metal tolerance of a leguminous plant such as alfalfa by sequestering the metals or changing their forms in the soil (Teng et al., 2011). The fixation of nitrogen by rhizobia also improves the plant’s resistance to metal stress by increasing soil fertility (Fagorzi et al., 2018). This is a unique advantage of the joint symbiosis between leguminous plants and rhizobia to mitigate heavy metal pollution in soil.

Recent research on different types of legume-rhizobia symbiosis systems has mainly focused on: (1) isolation of heavy metal-tolerant rhizobia and screening for plant growth-promoting traits (Wani and Khan, 2013; Fan et al., 2018), (2) mechanisms of resistance of rhizobia to heavy metals (Adediran et al., 2015; Nocelli et al., 2016), (3) screening for legumes that are tolerant to heavy metals (Abdelkrim et al., 2018), and (4) evaluation of the ecological remediation effect of legume-rhizobia symbiosis systems on heavy metal pollution (Ju et al., 2015; Shen et al., 2019). Because the number of symbiotic remediation systems that have been studied and tested is very limited, the diversity of rhizobial populations offers great opportunities for discovering high quality strains that can be used for bioremediation of heavy metal-contaminated soils. However, resources for rhizobia-legume nitrogen fixation systems with high efficiency are still lacking, especially those from some extreme environments.

Previous studies have found that there was abundant growth-promoting bacteria, such as rhizobia, in the VTM tailings (Yu et al., 2014). Culturable *Bradurhizobium* genus aymbiotic with *P. pinnata* was also isolated from the VTM tailings, and then a aymbiotic bioremedition system of *P. pinnata* and rhizobia was established for ecological remediation of the VTM tailings (Yu et al., 2017a). High-throughput sequencing technology found some other genus of rhizobia in the VTM tailing (Yu et al., 2019). It was hypothesis that there are more genus of rhizobia symbiotic with *P. pinnata*, and these rhizobia could show high nitrogen-fixing capacity and strong heavy metal tolerance, which would provide more high quality rhizobia resources for bioremediation of the VTM tailings or other heavy metal-contaminated soil. So, this study more comprehensively understand diversity, nitrogen-fixing capacity and heavy metal tolerance of culturable *P. pinnata* rhizobia in the VTM tailings, providing a basis for the development and utilization of rhizobia.

## Materials and methods

### Soil collection and trapping of rhizobia

Soil samples were collected from a VTM tailings area located in Panzhihua, Sichuan Province, China (101°58′10.89″E, 26°36′59.47 N) for compositional analysis and a *P. pinnata* pot experiment. The seeds of *P. pinnata* were collected in a mangrove forest in Wenchang, Hainan Province, China (110°47′E, 19°37’N). The mature seeds of *P. pinnata* were taken to laboratory and planted in pots containing VTM tailings. The pots were kept in a greenhouse with a day temperature of 25°C for 16 h and a night temperature of 17°C for 8 h. The potted trees were irrigated using tap water when needed. Three months later, when there were some big and pink nodules on the roots of *P. pinnata*, the plants were uprooted.

### Analysis of soil physicochemical properties and metal contents

Total nitrogen (N) and available N of the soil samples were determined using the Kjeldahl method and alkali N-proliferation method, respectively (Wang et al., 2016; Spargo and Alley, 2018). Soil pH, organic matter, available phosphorus (P) and potassium (K), were determined using the ASI method. Tailings samples were digested with a mixture of HNO_3_:HF:HCl (3:1:1 by vol) for the measurement of total metals, and the available metals in tailings were extracted by using 1 M C_2_H_7_NO_2_ and 0.2 M ethylenediaminetetraacetic acid (EDTA; Saadani et al., 2016). A soil sample known available metal content was designed in all the steps of available metal extraction and measurement process as quality control. Then, the concentration of total metals and available metals of iron (Fe), titanium (Ti), vanadium (V), chromium (Cr), manganese (Mn), zinc (Zn), copper (Cu), nickel (Ni), lead (Pb), and cadmium (Cd) were quantitated using inductively coupled plasma atomic emission spectrometry (ICP- AES) (IRIS IntrepidII, Thermo Electron Corporation, USA; Zhang et al., 2010).

### Isolation and purification of rhizobia

The fresh, big and pink nodules removed from the roots of *P. pinnata* were sterilized by soaking in 95% ethanol for 1 min, then in 0.1% HgCl_2_ for 3 min, and washed several times with sterile water. Under aseptic conditions, the surface-sterilized nodules were crushed in a sterilized EP tube, and a small aliquot of the nodule suspension was taken for steaking on Congo red-containing yeast mannitol agar (YMA, 10.0 g/L mannitol, 1.0 g/L yeast extract, 0.5 g/L K_2_HPO_4_·3H_2_O, 0.2 g/LMgSO_4_·7H_2_O, 0.1 g/L NaCl, and 1.0 g/L CaCO_3_ and 0.04 g/L Congo red, pH 7.0–7.2; Zevenhuizen et al., 1986; Sierra et al., 1999). Mucoid and white colonies were selected for repeated re-streaking until single colonies with uniform colony characteristics were observed (Brenner et al., 2005). Purification of rhizobia was confirmed by Gram staining and microscopic examination, and Gram-negative strains were kept for molecular identification (Brooks et al., 2016).

### Identification and phylogenetic analysis of rhizobia

Total DNA was extracted from purified isolates using the phenol-chloroform method (Chang et al., 2011), and 16S ribosomal RNA (rRNA), BOXA1R and four housekeeping genes (*atpD, recA, rpoB, glnII*) were amplified by PCR as described (Vinuesa et al., 2008; Menna et al., 2009). The PCR products were sequenced by Sangon Biotech (Shanghai, China), and the sequences were submitted to GenBank for assignment of accession numbers. Similarity analysis was performed by searching using BLAST in GenBank. Neighbor-joining (NJ) trees of the 16S rRNA and housekeeping genes were constructed using MEGA7.0 software with bootstrap values of 1,000 replicates (Menna et al., 2009). Sequence data for the four housekeeping genes were concatenated into a single *atpD-recA-rpoB-glnII* sequence for multilocus sequence analysis (MLSA; Vinuesa et al., 2008; Menna et al., 2009).

### Heavy metal tolerance tests of rhizobia

The resistance of the 20 isolated rhizobia to Ni, Cd, Mn, and Cu was assayed by measuring their growth in YMA liquid medium (3.0 g/L yeast extract, 5.0 g/L tryptone, 0.7 g/L CaCl_2_·2H_2_O, pH 7.0) containing different concentrations of metal ions by adding the salts of NiCl_2_, CdCl_2_, MnSO_4_, and CuSO_4_, respectively. The bacterial suspensions (50 μL, 10^8^ cells/mL) were inoculated into 5 mL YMA liquid medium. The medium without heavy metal was used as the control. The minimum inhibitory concentration (MIC) and lethal concentration (MLC) were obtained by measuring the optical density (OD_600nm_) of the bacterial cultures with a spectrophotometer (UV-3300; Shanghai MAPADA, Shanghai, China) after incubation in an orbital shaker (28°C, 150 rpm) for seven days (Mao et al., 2020). MLC was defined as the lowest concentration of metal ion in solid medium where the isolate growth was not observed, while MIC as the lowest concentration of metal ion in the solid medium where the isolate growth was weaker than that in the heavy metal-free control (Yu et al., 2014). The bacterial cultures were repeated three times for each treatment, and OD_600nm_ readings were taken in triplicate.

### Symbiotic nitrogen fixation capacity of rhizobia

After the amplification of *nif*H gene of the isolates, the PCR products were sequenced by Sangon Biotech (Shanghai, China), and the sequences were submitted to GenBank for assignment of accession numbers. Similarity analysis was performed using BLAST in GenBank. Neighbor-joining (NJ) trees of the *nif*H gene were constructed using MEGA7.0 software with a bootstrap value of 1,000 replicates (Menna et al., 2009).

**Figure 1:**
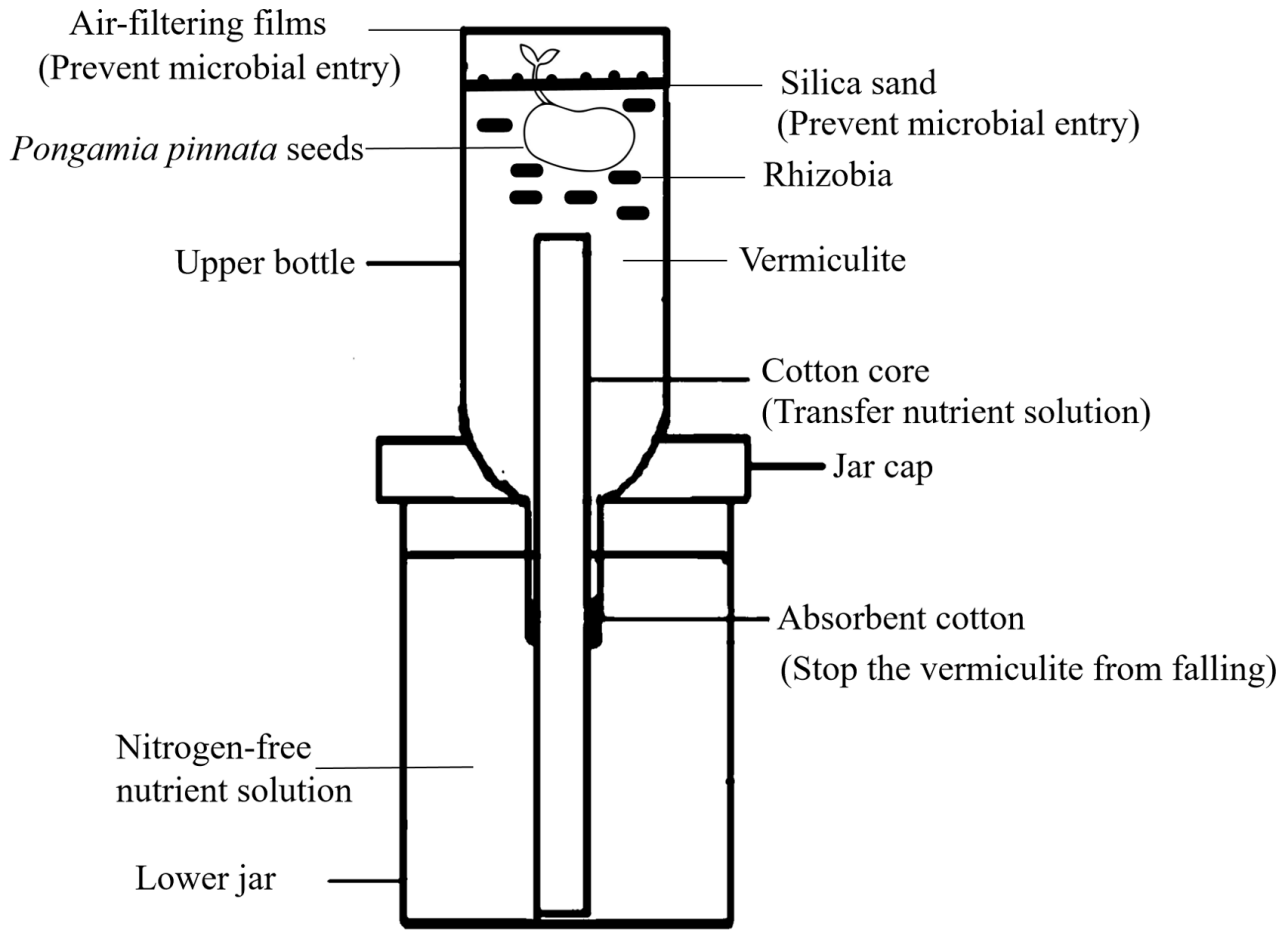
The diagram of potted experimental equipment.

To test the symbiotic nitrogen fixation capacity of the rhizobia isolates, some representative strains of different genera rhizobia were selected for rhizobia-*P. pinnata* pot experiment by using the potted experimental equipment (Figure 1). Leonard jars, which consisted of two parts, i.e., an upper bottle and a lower jar, were assembled as the apparatus for testing nitrogen fixation activities of rhizobia (Yu et al., 2017a). The lower jar contained nitrogen-free nutrient solution, while the upper bottle was filled with vermiculite as the substrate. The assembled Leonard jars were autoclaved (100 KPa, 121°C) for 30 min after covering the upper bottles with air-filtering films. Some mature and plump seeds of *P. pinnata* were selected for surface sterilizing using diluted NaClO_3_ and ethanol. The seeds were sowed in the upper bottles after germination, and then the air-filtering films were used to cover the upper bottles again. Approximately 3 × 10^8^ of fresh rhizobia cells were inoculated around the rhizosphere of a *P. pinnata* seedling, and then the sterilized silica sand was used to cover the vermiculite to avoid contamination. *P. pinnata* trees in the non-inoculation pots was designed as the control (CK), and each treatment repeated three times. The pots were kept in a greenhouse with a day temperature of 25°C for 16 h and a night temperature of 17°C for 8 h. After 6 months, *P. pinnata* plants were uprooted, and the nodule numbers, plant height, root length, biomass (dry weight) and nitrogen content were measured using the previous methods (Yu et al., 2017a).

### Statistical analysis

The experimental data were averaged out of at least three independent replicates for each treatment. Microsoft Excel 2016 was used to calculate means and standard deviations. IBM SPSS Statistics 26.0 was used to perform Tukey’s test at *p* < 0.05.

## Results

### Physicochemical properties and metal content of soil samples

To establish the basic characteristics of the VTM tailings, the physicochemical properties and metal contents were measured for the collected soil samples (Table 1). Soil pH was slightly acidic with a value of 5.77 ± 0.15. The contents of soil organic matter and total nitrogen were very low in the VTM tailings. The available N, P, and K in the VTM tailings accounted for 23.2, 15.2, and 24.9% of the total contents, respectively. The N, P, and K content in the VTM tailings was far lower than the cultivated soil nitrogen content, so the VTM tailings is very barren. As three main components of the VTM tailings, the concentrations of total Ti and Fe were very high as expected at 108 g/kg and 104 g/kg, respectively, while the content of total V (952.3 mg/kg) was relatively low. Interestingly, the concentration of total Mn reached 3,239.20 mg/kg, and the contents of total Ni, Zn, and Cu were more than 100 mg/kg. Only the amounts of total Cr, Pb, and Cd were relatively small in the VTM tailings. The available Ti, Cd, and Cr in tailings were not detected, the content of available V and Zn was very low, and the available Fe was highest, following by Cu, Mn and Ni.

**Table 1:** Physicochemical properties and metal contents of the VTM tailings.

Property	Average value	Property	Average value
pH	5.77 ± 0.15	Organic matter (‰)	16.98 ± 4.45
Total N (mg/kg)	58.80 ± 1.70	Available N (mg/kg)	13.64 ± 3.03
Total P (mg/kg)	86.58 ± 4.95	Available P (mg/kg)	13.13 ± 0.88
Total K (mg/kg)	54.99 ± 1.55	Available K (mg/kg)	13.71 ± 1.86
Total Fe (mg/kg)	103972.67 ± 2715.20	Available Fe (mg/kg)	76.67 ± 1.00
Total Ti (mg/kg)	107864.00 ± 2112.42	Available Ti (mg/kg)	0
Total V (mg/kg)	952.3 ± 342.24	Available V (mg/kg)	0.41 ± 0.05
Total Mn (mg/kg)	3239.20 ± 122.08	Available Mn (mg/kg)	10.85 ± 1.42
Total Ni (mg/kg)	527.04 ± 12.23	Available Ni (mg/kg)	9.57 ± 0.96
Total Zn (mg/kg)	287.45 ± 26.52	Available Zn (mg/kg)	0.41 ± 0.03
Total Cu (mg/kg)	191.49 ± 17.78	Available Cu (mg/kg)	14.59 ± 1.16
Total Cr (mg/kg)	98.51 ± 5.20	Available Cr (mg/kg)	0
Total Pb (mg/kg)	27.41 ± 3.51	Available Pb (mg/kg)	3.88 ± 0.12
Total Cd (mg/kg)	23.18 ± 2.49	Available Cd (mg/kg)	0

### Rhizobia isolates and BOXA1R-PCR fingerprints analysis

**Figure 2:**
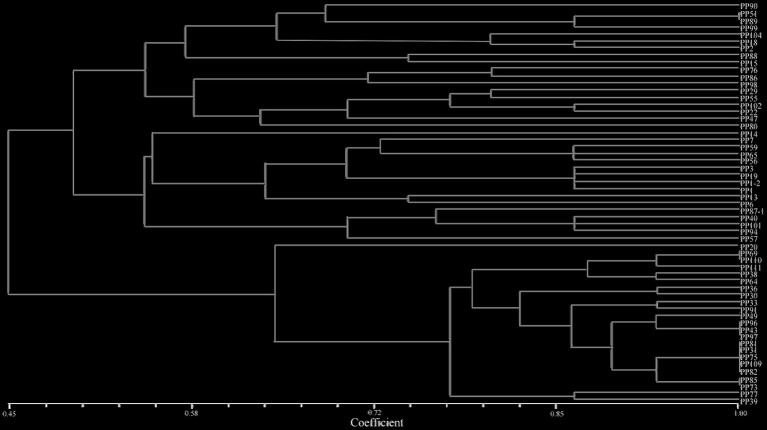
BOX-A1R dendrogram for 56 rhizobia isolated from the nodules of *Pongamia pinnata* in VTM mine tailings.

The results showed that *P. pinnata* can grow well in VTM tailings. A total of 57 rhizobia strains with the characteristic white mucoid colonies, Gram-negative and rod-shape features were isolated from the nodules of *P. pinnata* growing in the VTM tailings. The similarities among the 57 isolates ranged from 0.45 to 1.00 in the BOX A1R-PCR fingerprint dendrogram, including 49 distinct fingerprint patterns (Figure 2). These strains were clustered into two groups at 45% similarity level, three groups at 49% similarity level, 8 groups at 61% similarity, 18 groups at 74% similarity, and 41 groups at 91% similarity level. There were also some strains on the same branch with 100% similarity, such as PP31, PP75, PP81, PP82, and PP109.

### Heavy metal tolerance of rhizobia

According to the results of BOXA1R-PCR fingerprints analysis (Figure 2), eight isolates with 100% similarity were deleted, and 49 strains were kept for the determination of heavy metal tolerance. Among them, 20 strains showed different levels of tolerance against four heavy metals including Cu, Ni, Mn, and Zn. After culturing in YMA medium with Cu^2+^ for 7 days, the survival rates were 85% at 100 mg/kg, 20% at 200 mg/kg, 10% at 300 mg/kg, and 5% at 400 mg/kg. For nickel (Ni^2+^), they were 45% at 100 mg/kg, 20% at 300 mg/kg, 10% at 500 mg/kg, and 5% at 700 mg/kg. For cadmium (Cd^2+^), they were 55% at 200 mg/kg, 30% at 400 mg/kg, 15% at 600 mg/kg, and 10% at 800 mg/kg. For manganese (Mn^2+^), it was 75% at 500 mg/kg, 65% at 1,300 mg/kg, 20% at 2100 mg/kg, and 10% at 2900 mg/kg.

**Figure 3:**
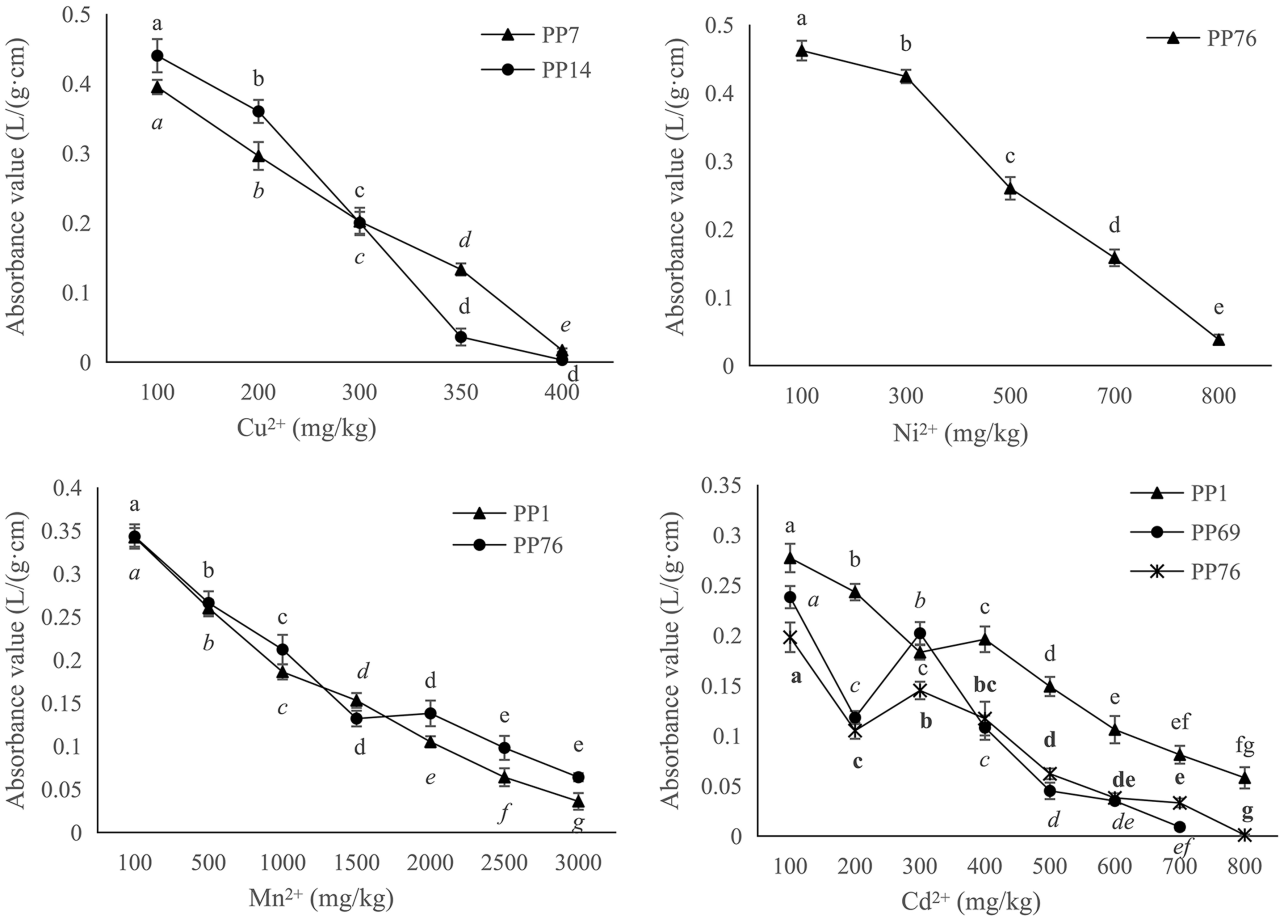
Growth curves for the five rhizobia strains in different concentration of heavy metal.

Only five rhizobia strains (PP1, PP7, PP14, PP69, and PP76) showed relatively higher tolerance to the four heavy metals (Table 2; Figure 3). PP76 tolerated against Ni, Cd, and Mn with an MIC at 100, 200, 300 mg/L, respectively, and with an MLC at 600, 800, 3,200 mg/L, respectively. PP1 showed high tolerance to Cd and Mn with an MIC at 200, 300 mg/L, respectively, and an MLC at 900, 3100 mg/L, respectively. Strains PP7 and PP14 only showed tolerance against Cu with an MIC at 100 mg/L, and an MLC at 400 and 350 mg/L, respectively. Only PP69 showed high tolerance to Cd with an MIC at 100 mg/L and an MLC at 700 mg/L.

**Table 2:** Heavy metal minimum inhibitory concentration (MIC) and lethal concentration (LC) of rhizobia strains from the VTM tailings.

Rhizobia	Heavy metals	MIC (mg/L)	LC (mg/L)
PP76	Ni	100 ± 14.43c	600 ± 12.22d
	Cd	200 ± 8.08b	800 ± 0.58bc
	Mn	300 ± 13.50a	3,200 ± 4.70a
PP1	Cd	200 ± 8.19b	900 ± 6.00b
	Mn	300 ± 9.54a	3,100 ± 9.64a
PP7	Cu	100 ± 23.80c	400 ± 2.00e
PP14	Cu	100 ± 10.30c	350 ± 2.60e
PP69	Ni	100 ± 11.06c	700 ± 3.06 cd

### Identification of rhizobia

**Figure 4:**
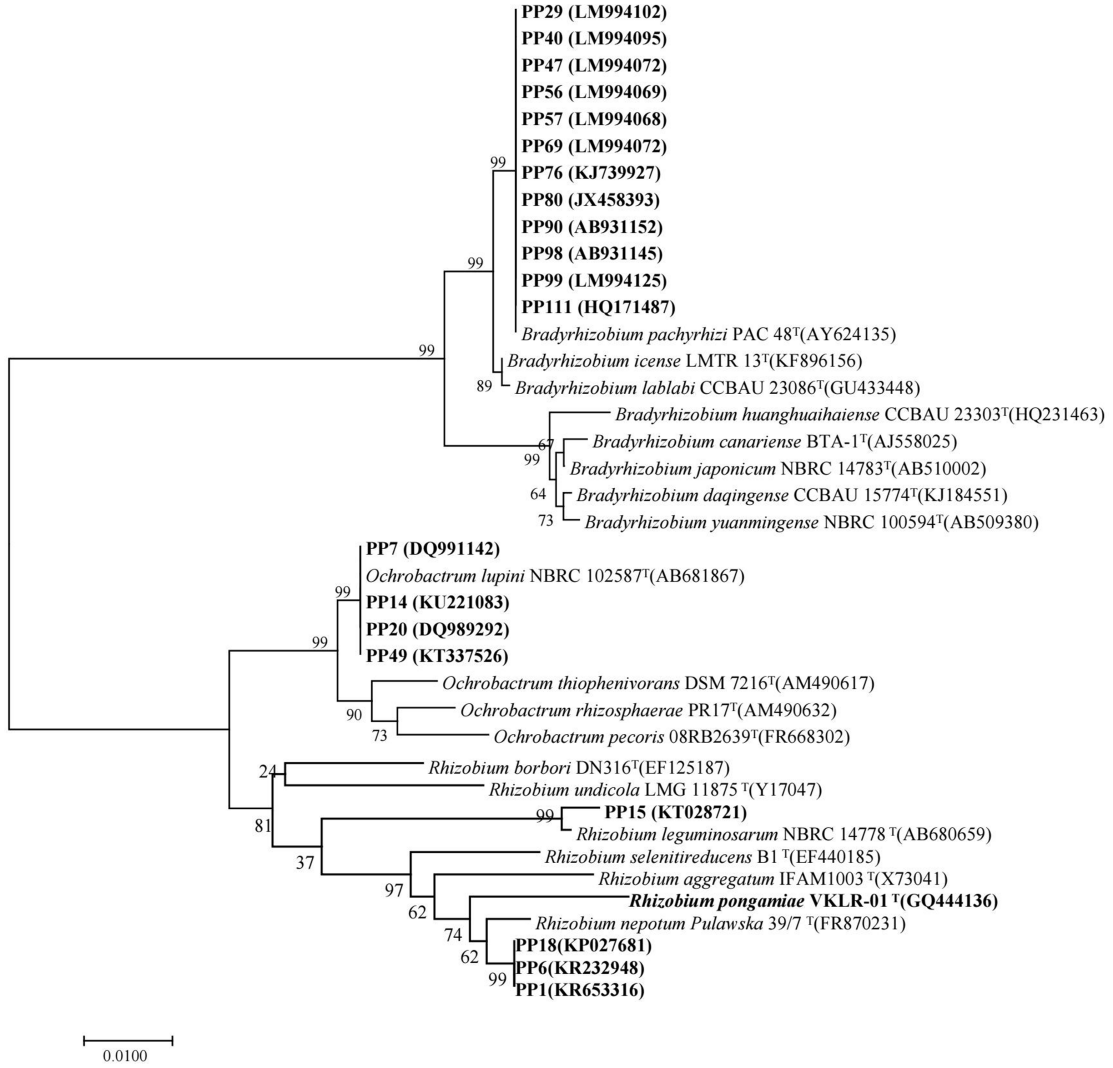
Phylogenetic tree of the rhizobial 16S rRNA genes. The scale bar corresponds to 0.01 substitutions per nucleotide position. The sequence numbers in GenBank are presented in the following parentheses. Superscript “T” means type stains.

The 20 strains with heavy metal tolerance were selected for molecular identification by 16S rRNA gene sequencing. The sequences of 16S rRNA genes were obtained and compared using BLAST in the National Center for Biotechnology Information (NCBI) database. Similarity analysis of 16S rRNA genes showed that the 20 rhizobia strains were classified into three different genera: *Bradyrhizobium* (12 strains), *Ochrobactrum* (4 strains) and *Rhizobium* (4 strains). The phylogenetic tree of the 16S rRNA gene also divided the 20 rhizobia strains into three different branches (Figure 4). Among them, 12 *Bradyrhizobium* isolates (PP29, PP40, PP47, PP56, PP57, PP69, PP76, PP80, PP90, PP98, PP99, and PP111) were closest to *Bradyrhizobium pachyrhizi* PAC 48^T^ with 100% similarity under the same branch (Figure 4). Three *Rhizobium* isolates (PP1, PP6 and PP18) were 99.12% similar with *Rhizobium nepotum* Pulawska 39/7^T^, whereas *Rhizobium* sp. PP15 was most closely related to *Rhizobium leguminosarum* NBRC14778^T^ with 99.47% similarity. Other four isolates (PP7, PP14, PP20, and PP49) were classified into *Ochrobactrum*, which was 100% similar with *Ochrobactrum lupini* NBRC 102587^T^ under the same branch (Figure 4).

### Phylogenetic analysis of rhizobia

**Figure 5:**
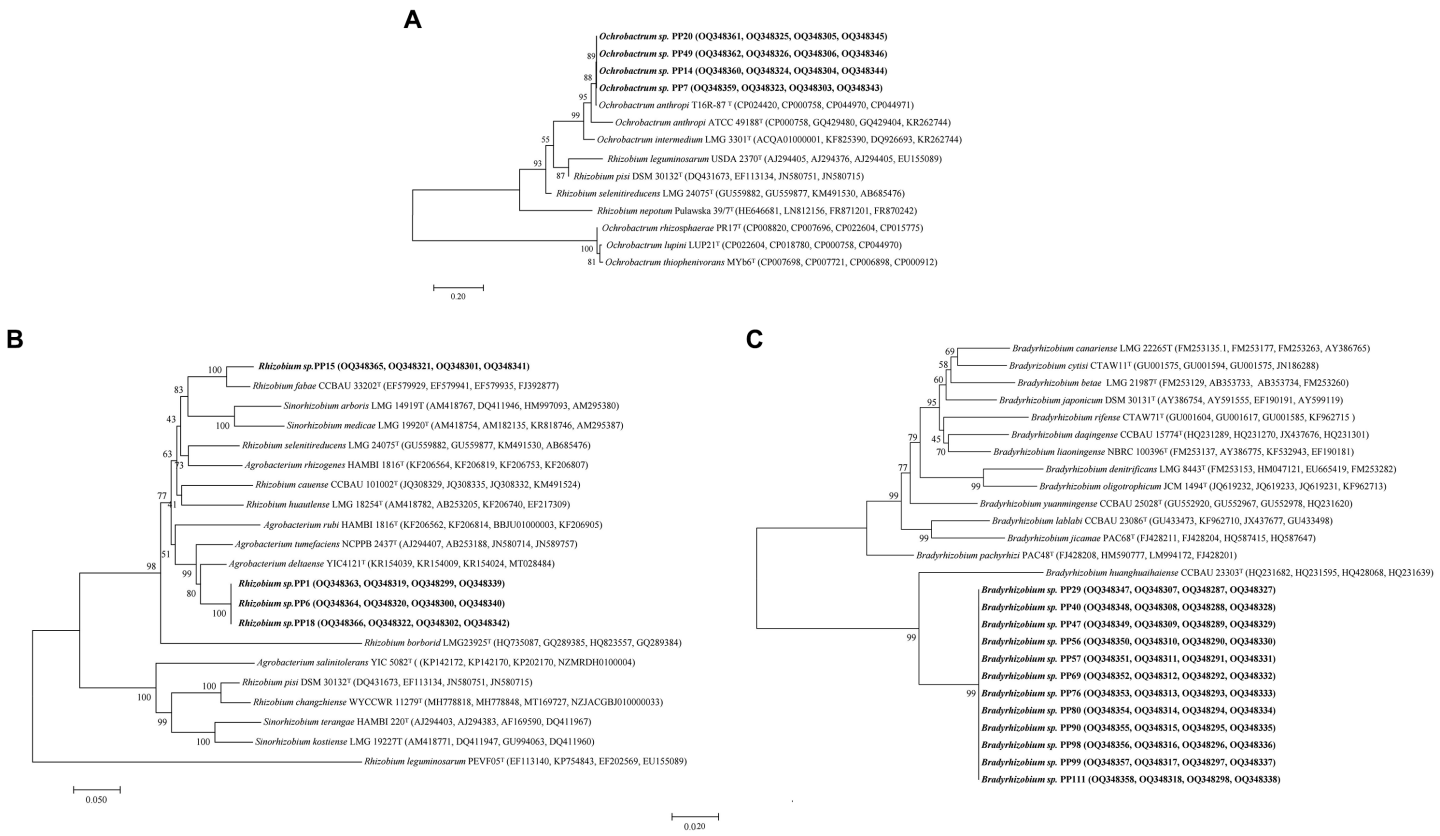
Phylogenetic tree of the concatenated housekeeping genes (*atpD-glnII*-*recA*-*rpoB*) for *Ochrobactrum* **(A)**, *Rhizobium* **(B)**, and *Bradyrhizobium* **(C)** genus.

To accurately determine phylogenetic status of the 20 rhizobia, amplified the four house-keeping genes (*atpD*, *recA*, *rpoB* and *glnII*) of the isolates and built a phylogenetic tree for each genus. For 12 *Bradyrhizobium* isolates, sequences of the house-keeping genes [*atpD* (407 bp), *recA* (380 bp), *rpoB* (522 bp), and *glnII* (474 bp)] were used to perform the multi-locus sequence analysis (MLSA) by constructing a longer housekeeping gene fragment (1,783 bp). Then, the phylogenetic tree of these 12 *Bradyrhizobium* isolates was built using neighbor-joining method (Figure 5C). For 4 *Rhizobium* isolates, sequences of the house-keeping genes [*atpD* (298 bp), *recA* (246 bp), *rpoB* (554 bp) and *glnII* (339 bp)] were subjected to multi-locus sequence analysis (MLSA) by constructing a longer housekeeping gene fragment (1,437 bp). Then, the phylogenetic tree of these 4 *Rhizobium* isolates was built using neighbor-joining method (Figure 5B). For 4 *Ochrobactrum* isolates, sequences of the four house-keeping genes [*atpD* (387 bp), *recA* (385 bp), *rpoB* (412 bp) and *glnII* (447 bp)] were subjected to multi-locus sequence analysis (MLSA) by constructing an longer housekeeping gene fragment (1,631 bp) (Figure 5A).

The MLSA phylogenetic tree of the four housekeeping genes at genus level was basically the same as that of the 16S rRNA gene, but there were some differences at species level. Twelve *Bradyrhizobium* isolates were most closely related to *Bradyrhizobium huanghuaihaiense* CCBAU 23303^T^ with 99.24% similarity, and *Bradyrhizobium pachyrhizi* PAC 48^T^ with 98.70% similarity. The isolate *Rhizobium* sp. PP15 was closest to *Rhizobium fabae* CCBAU 33202^T^ with 99.35% similarity, and *Rhizobium pisi* DSM 30132^T^ with 98.55% similarity. Other three *Rhizobium* isolates (PP1, PP6, and PP18) were closest to *Agrobacterium deltaense* YIC4121^T^ and *Agrobacterium tumefaciens* NCPPB 2437^T^ with 98.95 and 98.44% similarity, respectively. Four *Ochrobactrum* strains were closest to *Ochrobactrum anthropi* T16R-87^T^and *Ochrobactrum lupini* LUP21^T^ with 99.74 and 97.83% similarity, respectively.

### Symbiotic nitrogen fixation capacity of rhizobia

**Figure 6:**
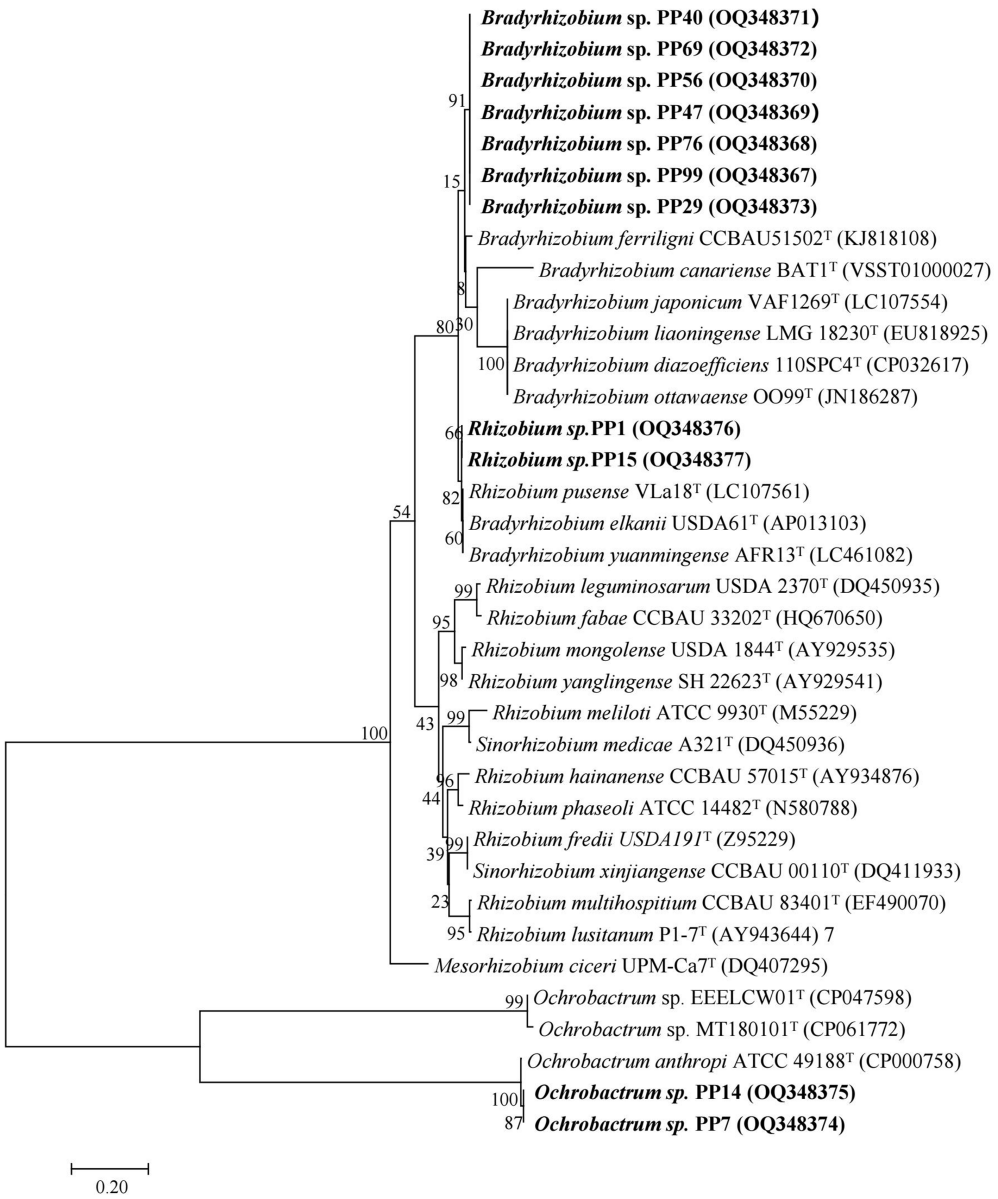
Phylogenetic tree of rhizobial *nif*H genes. The scale bar corresponds to 0.02 substitutions per nucleotide position.

We amplified the *nif*H gene of the isolates and built a phylogenetic tree using neighbor-joining method (Figure 6). As was similar with the trees of 16S rRNA genes and the house-keeping genes, the same genus was clustered together. Four *Bradyrhizobium* isolates were most closely related to *Bradyrhizobium ferriligni* CCBAU51502^T^, two *Rhizobium* isolates were most closely related to *Rhizobium pusense* VLa18^T^, and two *Ochrobactrum* isolates were most closely related to *Ochrobactrum anthropi* ATCC 49188^T^.

The 20 representative rhizobia with heavy metal tolerance were selected to determine their capacity of symbiotic nitrogen fixation using *P. pinnata* pot experiment. When the 20 rhizobia were, respectively, inoculated around the *P. pinnata* rhizosphere, only 11 strains built symbiotic relationships with *P. pinnata*. Moreover, the nodule numbers of *P. pinnata* inoculated with different rhizobia strains were fully diverse (Table 3). Plants inoculated with *Rhizobium* produced the highest number of roots nodules, suggesting *Rhizobium* had the strongest nodulating capability for *P. pinnata*, *Bradyrhizobium* was the next, and *Ochrobactrum* was the weakest at nodulation. In all treatments, more nitrogen content in *P. pinnata* was found in the aboveground parts than in the roots. The nitrogen content in the inoculated plants was significantly (*p* < 0.05) higher than that in the non-inoculated control. Except for *Bradyrhizobium* sp. PP76 and PP69, the trend of other rhizobia strains’ nitrogen fixation capacity was similar to that of symbiotic nodule number with the following order: *Rhizobium* > *Bradyrhizobium* > *Ochrobactrum*. Among them, *Rhizobium* sp. PP1 showed the strongest nitrogen fixation activity, and the nitrogen content of the plants’ aboveground parts and roots was 2.4–1.8 times that of the non-inoculation control.

**Table 3:** Nitrogen content, biomass, and growth of *Pongamia pinnata* inoculated with different rhizobia strains.

Strain	Genus	Nodule number	Plant height (cm)	Root length (cm)	Biomass (g/plant)	Nitrogen content (g/kg)	
						Aboveground part	Underground part
CK	-	0 ± 0 g	14.03 ± 1.76e	28.67 ± 1.80 h	4.03 ± 0.64e	15.82 ± 0.71e	12.28 ± 1.34e
PP1	*Rhizobium*	141 ± 6a	30.47 ± 2.06b	99.97 ± 6.28bc	16.04 ± 1.67ab	38.58 ± 0.42a	22.04 ± 0.20a
PP15	*Rhizobium*	132 ± 5a	34.33 ± 3.54ab	85.10 ± 3.53d	17.61 ± 2.27a	33.73 ± 2.26b	20.98 ± 0.26a
PP56	*Bradyrhizobium*	97 ± 7b	22.93 ± 2.23 cd	120.33 ± 3.35a	10.41 ± 1.17bcd	30.94 ± 1.66b	19.99 ± 0.15a
PP69	*Bradyrhizobium*	60 ± 6c	31.13 ± 4.15b	108.70 ± 8.82ab	10.63 ± 1.34bcd	31.92 ± 1.95b	13.87 ± 0.61de
PP76	*Bradyrhizobium*	41 ± 6d	33.30 ± 1.31b	87.53 ± 6.31 cd	14.84 ± 1.81ab	33.48 ± 1.20b	17.12 ± 0.47b
PP99	*Bradyrhizobium*	24 ± 5e	20.57 ± 1.5bc	36.57 ± 1.00gh	7.05 ± 0.52de	30.21 ± 1.32b	15.57 ± 0.26bcd
PP47	*Bradyrhizobium*	14 ± 2ef	34.27 ± 2.05ab	58.83 ± 3.69ef	8.64 ± 0.10cde	21.02 ± 0.85 cd	16.86 ± 1.41bc
PP29	*Bradyrhizobium*	6 ± 1 fg	31.47 ± 2.28b	52.07 ± 2.34ef	9.19 ± 1.99cde	33.48 ± 1.20b	17.12 ± 0.47b
PP40	*Bradyrhizobium*	2 ± 0 g	27.80 ± 2.65bc	46.90 ± 6.49 fg	13.93 ± 1.77abc	23.39 ± 1.68c	16.50 ± 0.76bc
PP7	*Ochrobactrum*	2 ± 1 fg	34.63 ± 0.32ab	64.30 ± 4.42e	11.20 ± 3.97bcd	17.37 ± 0.69de	14.43 ± 1.11cde
PP14	*Ochrobactrum*	2 ± 1 g	41.37 ± +2.28a	62.87 ± 2.79e	11.06 ± 2.16bcd	19.54 ± 3.18cde	15.36 ± 1.26bcd

Data are given as mean ± SD. Lowercase letters following each set of data in the same row indicate significant difference at *p* < 0.05 according to Tukey’s test.

Except for nitrogen fixation capacity, these rhizobia strains showed different levels of plant growth promoting activities for *P. pinnata*. Among them, the plant height, root length and biomass of the non-inoculated control were the lowest. The plant height of *P. pinnata* inoculated with *Ochrobactrum* sp. PP14 was the highest, whereas the root length was the lowest, just as in the non-inoculated control. Compared with the non-inoculated control plants, the improved height of the inoculated plants ranged from 47 to 195%, while the increase of root length of the inoculated plants ranged from 119 to 320%. Because of the promoting effect of rhizobia, the biomass of the inoculated *P. pinnata* plants was significantly (*p* < 0.05) higher than that of the control plants. Compared with the non-inoculated plants, the biomass of the inoculated treatments was improved by 1.75–4.27 times. The biomass improvement of *P. pinnata* inoculated *Rhizobium* sp. PP15 was the highest among all of them.

## Discussion

### Physicochemical properties and metal contaminations in the VTM tailings

The quality of the soil depends on its physicochemical properties. Soil pH affects soil microbial diversity and function (Blum et al., 2018), and the pH of VTM tailings in Sichuan Province, China, was found to be quite acidic, which is similar to the soil near a zinc blende mine north of Spain (Pérez-Esteban et al., 2014). Thus, the microbial communities in the VTM tailings must be more adaptable to an acidic environment. The available N, P, K and organic matter were as low as in other mine tailings (e.g., in Mexico), which indicated that the VTM tailings was not very fertile (Armienta et al., 2019). The Ti concentration was up to 38 times higher than that in the similar soil near a Ti mining site in Kenya, and the V concentration was up to 7.5 times higher than that in Cuban soils on average (Alfaro et al., 2014; Maina et al., 2016). The concentration of Fe was approximately 3,000 mg/kg, which is higher than the soil near a steel plant in India (Kaur et al., 2019). Compared to the soil near a coal mine in China with severe Cu, Zn, and Cr pollution, the concentration of these elements in Sichuan VTM tailings was 6.70, 3.69, and 1.54 times higher, respectively; but Pb was lower at 1.45 mg/kg (Liu et al., 2020). The concentration of Mn and Ni in VTM tailings was approximately 5 and 3 times higher, respectively, than in the magnetite tailings after growth of *Imperata cylindrica* (Yuan et al., 2018). The Cd concentration already exceeded the minimum inhibitory concentration for plant growth (Zhang F. et al., 2019). Consequently, the reason why plants grown in the VTM soil were infertile may be due to the high heavy metal contents and low available N, P, K, and organic matter. Therefore, when carrying out ecological restoration, attention must be paid to reducing the concentration of heavy metals in the soil and increasing the content of nutrients.

### Diversity and phylogeny of *Pongamia pinnata* rhizobia in the VTM tailings

As an biofules resource, *P. pinnata* is a fast-growing leguminous tree with the potential for high oil seed production and can grow on marginal land (Scott et al., 2008). Only two genera of *Rhizobium* genera including *R. pongamiae, R. miluonense*, and *Bradyrhizobium* genera including *B. liaoningense, B. elkanii*, *B. yuanmingense* were found to be symbiotic nitrogen fixation with *P. pinnata* in India and Australia (Rasul et al., 2012; Arpiwi et al., 2013; Kesari et al., 2013). However, three genera rhizobia of *Rhizobium*, *Bradyrhizobium* and *Ochrobactrum* symbiotic with *P. pinnata* were isolated from the VTM tailings, which revealed there were abundant rhizobia in the VTM tailings. These rhizobia included *B. pachyrhizi*, *R. nepotum*, *R. nepotum*, and *O. lupini*, indicating *P. pinnata* rhizobia isolated from the VTM tailings were different form previous reported others. So, the VTM tailings was a resource pool including abundant functional microbiology. Although it was the first time that *Ochrobactrum* was found to have symbiotic nodulation with *P. pinnata*, their symbiotic nitrogen fixation efficiency were not high (Table 2).

The distribution of the *Rhizobium* population can easily be changed by the influence of different environmental factors (Stefan et al., 2018). Because of multiple heavy metal pollution and barren environmental factors, rhizobia symbiotic with *P. pinnata* for the VTM tailings were different from others. The proportion of *Bradyrhizobium* strains was highest among three rhizobia genera in the VTM tailings, probably because of stronger resistance of *Bradyrhizobium* to heavy metals. Compared with *R. pongamiae* VKLR-01 isolated from root nodules of *P. pinnata,* their genetic similarity is not high (Kesari et al., 2013). Some of the strains had specific genetic traits which helped to enhance their adaptability to the toxic environment of heavy metal ions. These special rhizobia from the VTM tailings also proved that microbial composition in a rhizobial system has host-specific and bio-geographical distribution characteristics (Zhang et al., 2011).

### Heavy metal tolerance of *Pongamia pinnata* rhizobia in the VTM tailings

Although there are multiple heavy metal pollutants and very poor nutrition in there VTM tailings, abundant heavy metal resistant and plant growth promoting bacteria survival in the extreme environment (Yu et al., 2014). Because of the extreme heavy metal environment, *P. innata* rhizobia from the VTM tailings also showed heavy metal resistance. Environmental factors following the order: soil pH > heavy metals > nitrogen > soil texture had distinct impacts on microbial community (Deng et al., 2018), indicating that heavy metals were very important affection factor for soil microbe. Only 40% rhizobia from the VTM tailings showed tolerance against Cu, Ni, Mn, and Zn. The tolerance concentrations of these metals (except for Mn) for these strains were higher than those in the VTM tailings, and different isolates had different level of tolerance to heavy metals, indicating soil heavy metals are not the only factor affecting strain resistance. Some microbes metabolize and transform heavy metal into a less hazardous form for surviving in such harsh environments, resulting in the formation of heavy-metal-resistant microbes (Prabhakaran et al., 2016), so these microbes had their own unique resistance characteristics. From BOXA1R-PCR fingerprints and Phylogenetic characteristic, rhizobia form the VTM tailings had different genotype, so their tolerance to heavy metals was different, and even some isolates did not showed tolerance to the four tested heavy metals.

In other research on rhizobial systems, most bacteria were only tolerant to a single heavy metal and only a few were resistant to multiple types of heavy metals (Stan et al., 2011; Fan et al., 2018), which was consistent with the tolerance to heavy metals of rhizobia from the VTM tailings. *Bradyrhizobium* sp. PP76 was resistant to Ni, Cd, Mn of the four metal ions and *Rhizobium* sp. PP1 was resistant to Cd and Mn, which makes them the best choices for the establishment of symbiotic systems of leguminous plants and rhizobia in heavy metal-contaminated soils.

### Nitrogen fixation capacity of *Pongamia pinnata* rhizobia in the VTM tailings

As a typical function of rhizobia, symbiotic nitrogen fixation is relation to *nod*, *nif* and *fix* genes, such as *nifH* named as dinitrogenase reductase (Shamseldin, 2013). The *nifH* gene is the biomarker most widely used to study the ecology and evolution of nitrogen-fixing bacteria (Gaby and Buckley, 2014), so the amplified *nif*H gene was an initial evidence of the nitrogen-fixing capability of the rhizobia isolates from the VTM tailing. From the phylogenetic tree, the *nifH* genes of three genera rhizobia were also consistent with the 16S rRNA and house-keeping genes with high similarity among the same genus rhizobia. Because symbiotic nitrogen fixation was decided by series of *nif* genes, e.g., *S. meliloti* and *R. leguminosarum* bv. viciae have a restricted set of 9 and 8 nif genes, respectively (Masson-Boivin et al., 2009). Therefore, although the *nifH* genes of same genus with high genotype similarity was on the same branch, these rhizbia showed different nitrogen fixation and plant growth capacity. These rhizobia had symbiotic N-fixation ability with the leguminous host plant of *P. pinnata* and were facilitative in promoting plant growth. Almost of rhizobia showed consistence between symbiotic nitrogen fixation and plant biomass, except for *Ochrobactrum* sp. PP7 and PP14. Although *Ochrobactrum* sp. PP7 and PP14 showed lowest symbiotic nitrogen fixation efficiency among the eleven isolates, their plant-growth promoting activity was stronger than some *Bradyrhizboium* sp. isolates, indicating that *Ochrobactrum* sp. PP7 and PP14 might had some of other plant-growth promoting capacity (Yu et al., 2017b).

The excessive metal concentrations cause undeniable damage to rhizobia, legumes and their symbiosis to affect efficiency of symbiotic nitrogen fixation (Zahran, 1999; Ahmad et al., 2012), which does not hinder that rhizobia increase phytoremediation by nitrogen fixation and production of plant growth-promoting factors and phytohormones (Pajuelo et al., 2011). So, legume–rhizobium symbioses has been considered as a tool for bioremediation of heavy metal polluted soils (Pajuelo et al., 2011). However, rhizobium should have heavy-metal resistance to improve legume–rhizobium symbiosis in bioremediation of heavy metal polluted soil (El-Tahlawy and Ali, 2021). *P. pinnata* rhizobia from the VTM tailings did not only show nitrogen fixation capacity but also heavy metal tolerances, so these rhizobia can be used to build symbiosis bioremediation system for heavy metals. *P. pinnata* inoculated with *B. liaoningense* PZHK1 was proved to show huge potential for phytoremediation of mine tailings, which had applied for soil and ecological remediation at the VTM tailings (Yu et al., 2017a, 2019). These rhizobium isolates with nitrogen fixation capacity and heavy metal resistance provided excellent microbial resources for bioremediation of the VTM tailings to other heavy metal polluted soil.

## Conclusion

The application of the symbiotic remediation systems of rhizobia and leguminous plants is a major research area with a focus on bioremediation of the multiple heavy metal-polluted environments. There are at least three genera of culturable rhizobia in symbiosis with *P. pinnata* in VTM tailings, namely, *Bradyrhizobium*, *Ochrobactrum*, and *Rhizobium*. Some rhizobia have high N-fixing efficiency, plant growth-promoting capacity, and resistance to heavy metals, indicating there are abundant functional microbial resources in extreme soil environment. Interestingly, the phenotype of strong N-fixing capacity seemed to coincide with the resistance to multiple metal ions, which could explain why *Bradyrhizobium* was the dominant genus of rhizobia around the *P. pinnata* rhizosphere in the soil contaminated with heavy metals. Because of resistance to several heavy metals, these isolates were competent candidates for the bioremediation of soils contaminated with multifarious metals. This study did not only reveal the genetic diversity and phylogeny of *P. pinnata* rhizobia in VTM tailings, but also provided important resources for the development of soil remediation techniques using rhizobium-legume symbiotic systems.

## Data availability statement

The data presented in the study are deposited in the GenBank repository, accession numbers OQ348361, OQ348325, OQ348205, OQ348345, OQ348362, OQ348362, OQ348306, OQ348346, OQ348360, OQ348324, OQ348304, OQ348344, OQ348359, OQ348323, OQ348303, OQ348343, OQ348365, OQ348321, OQ348301, OQ348341, OQ348363, OQ348319, OQ348299, OQ348339, OQ348364, OQ348320, OQ348300, OQ348340, OQ348366, OQ348322, OQ348302, OQ348342, OQ348347, OQ348307, OQ348287, OQ348327, OQ348348, OQ348308, OQ348288, OQ348328, OQ348349, OQ348350, OQ348351, OQ348352, OQ348353, OQ348354, OQ348355, OQ348356, OQ348357, OQ348358, OQ348309, OQ348310, OQ348311, OQ348312, OQ348313, OQ348314, OQ348315, OQ348316, OQ348317, OQ348318, OQ348287, OQ348288, OQ348289, OQ348290, OQ348291, OQ348292, OQ348293, OQ348294, OQ348295, OQ348296, OQ348297, OQ348298, OQ328327, OQ348328, OQ348329, OQ348330, OQ349331, OQ348332, OQ348333, OQ348334, OQ348335, OQ348336, OQ348337, OQ348338.

## Author contributions

TS, RJ, JY, and XY conceived research project, assayed rhizobia symbiotic nitrogen fixation capacity, performed statistical analysis, and drafted the manuscript. TS, XC, LZe, TZ, and XY collected soil samples and trapped rhizobia. RJ, XC, YG, LZo, KZ, and QX conducted general experiments, performed phylogenetic analysis, and identification of rhizobia. JY, LZo, and MM analyzed heavy metal tolerance of rhizobia. MM, SL, and TZ analyzed soil physicochemical properties and metal contents. XY and QC funded and supervised the experiments. All authors reviewed, edited, and approved the final manuscript.

## Funding

This research was supported by the National program on Key Research Project [2022YFD1901400], Demonstration Project of Transfer and Transformation of Scientific and Technological Achievements in Sichuan Province [2022ZHCG0030], and the Key Research Project of Deyang City [2022NZ015].

## Conflict of interest

The authors declare that the research was conducted in the absence of any commercial or financial relationships that could be construed as a potential conflict of interest.

## Publisher’s note

All claims expressed in this article are solely those of the authors and do not necessarily represent those of their affiliated organizations, or those of the publisher, the editors and the reviewers. Any product that may be evaluated in this article, or claim that may be made by its manufacturer, is not guaranteed or endorsed by the publisher.
